# Three-dimensional assisted techniques vs. conventional fixation in complex periarticular fractures: a systematic review of outcomes and complications

**DOI:** 10.3389/fbioe.2025.1725872

**Published:** 2025-12-16

**Authors:** Jinpeng Gong, Yanjun Sun, Yupeng Ma, Dong Han, Junbo Ge

**Affiliations:** 1 Department of Trauma Orthopedics, Yantaishan Hospital Affiliated to Binzhou Medical University, Yantai, China; 2 Operating Room, Yantaishan Hospital Affiliated to Binzhou Medical University, Yantai, China

**Keywords:** 3D printing, patient-specific implant, periarticular fracture, fracture fixation, surgicalplanning, orthopedic trauma

## Abstract

Complex periarticular fractures are difficult to manage as anatomical reduction and stable fixation using conventional methods are challenging. Patient-specific three-dimensional (3D) printed implants and surgical guides have been developed with recent technical advances in 3D printing technology. This study systematically evaluates 3D printing–assisted methods, including patient-specific implants surgical guides, and preoperative bone models, against traditional fixation techniques for periarticular fractures by examining intraoperative duration, fracture reduction quality, radiographic results, and functional outcomes and complication rates. The research team carried out a literature search on PubMed, Scopus, and Web of Science for clinical comparative studies published during the period of 2015–2025. The selected research evaluated the effectiveness of patient-specific 3D-printed implants and surgical guides as well as preoperative planning tools. Risk of bias (ROB) was assessed with ROBINS-I. Primary outcomes were collected and described. Seven studies (randomized controlled trials and cohort studies) fulfilled the inclusion criteria. Operative time and blood loss were significantly shorter in most of the studies using 3D-assisted techniques. Fracture reduction accuracy was improved in several studies with smaller articular step-off and higher rate of anatomical reduction. Functional outcome was similar in most of the studies with modest improvement reported in a few. Reduction in complications was seen or a similar rate in the 3D group. One study found no significant benefit of 3D guidance over conventional treatment. 3D-assisted fixation shows promise in enhancing surgical precision and efficiency in complex periarticular fractures. While early results are favorable, further high-quality research is needed to validate its long-term clinical benefits and cost-effectiveness.

## Graphical Abstract

**Figure:**
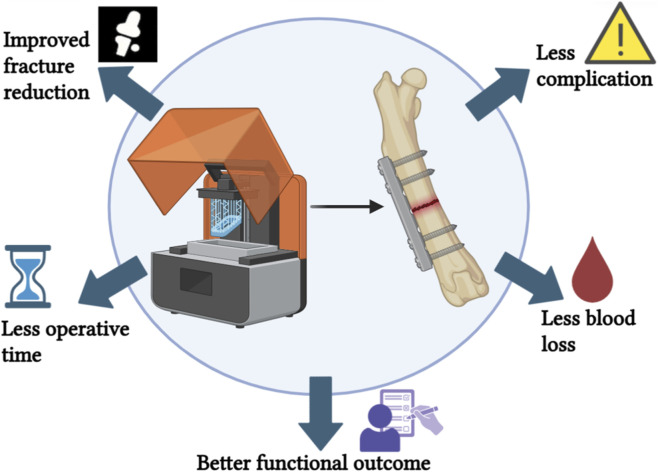

## 1 Introduction

Periarticular fractures occur frequently adjacent to joints (Dei Giudici et al., 2016). Types of complex periarticular fractures include breaks in the distal femur as well as the tibial plateau, acetabulum, and distal radius (Crowe and Kakar, 2023). These fractures pose treatment challenges because they demonstrate high-energy impact along with comminution which leads to joint incongruity and soft tissue damage causing difficulty in achieving anatomical reduction or stable fixation (Bertrand et al., 2015). Successful treatment results from precise surgical planning combined with anatomical joint reconstruction and stable fixation which support early movement (Crowe and Kakar, 2023; Rossi et al., 2025). Traditional fixation methods primarily utilize pre-contoured plates and intramedullary nails (Patel et al., 2024). Recent developments in implant design and intraoperative imaging have not fully addressed the difficulties presented by extremely fragmented fractures and patient-specific bone structures in traditional applications.

Orthopedic surgery has increasingly adopted additive manufacturing technologies over the recent years (Faghani-Eskandarkolaei et al., 2024). Its application to fracture fixation, particularly through the design and fabrication of patient-specific implants (PSIs), offers a paradigm shift from the conventional “one-size-fits-most” approach. Three-dimensional (3D) printing converts a computer-generated 3D image into a physical model (Balamurugan and Selvakumar, 2021). In this manuscript, the term “3D-assisted techniques” specifically refers to clinical applications of 3D printing technology, including PSI, patient-specific instrumentation (such as surgical guides), and 3D-printed bone models used for preoperative planning. By combining 3D printing with preoperative planning software like CT-based segmentation systems surgeons can precisely visualize and create customized surgical implants before surgery (Kim et al., 2021). It is particularly useful in complex cases where conventional implants may not be suitable or may not yield the best results due to anatomical variations or surgical considerations (Ariz et al., 2021). Since we now have multiple implants strategy design available for a given fracture, 3D printing could be used to select the optimal implant for the best patient outcome.

With several case reports, cohort studies, and clinical trials emerging, the potential benefits of 3D-printed PSIs for the treatment of complex periarticular fractures is beginning to be described (Tomaževič et al., 2021; Sattarahmady et al., 2024; Luenam et al., 2020; Chai et al., 2023). Reported benefits include improved operative efficiency (Bell et al., 2018), reduced surgical time (Ballard et al., 2020) and intraoperative blood loss (Prządka et al., 2025), and more anatomically congruent reconstructions (Moiduddin et al., 2023). PSIs demonstrate enhanced patient outcomes through better pain management, improved range of motion and earlier return to function (Einafshar et al., 2025). Several issues continue to affect 3D printing technology including high printing expenses and the requirement for specialized software training along with extended implant fabrication times (Willemsen et al., 2019). Available quantities of implants may be restricted by various factors and this limitation frequently occurs in acute trauma environments. The long-term safety and durability of implants continues to be an area of study with respect to mechanical fatigue challenges along with concerns about infection and hardware failure (Onică et al., 2024). The manufacturing process produces individualized implants that fit precisely with a patient’s bone structure by analyzing both bone shape and damage (Beheshtizadeh et al., 2022).

Currently, 3D-printed surgical guides and other patient-specific instruments are being increasingly applied in various orthopedic procedures. These include drill and screw guides for accurate trajectory control in acetabular, tibial plateau, and pelvic fixation; osteotomy and cutting guides for precise bone resections and deformity correction; and plate contouring templates that assist in pre-shaping standard implants for improved anatomical fit. In orthopedic oncology, resection and reconstruction guides help achieve accurate tumor margins and better alignment of grafts or custom implants. Collectively, these patient-specific tools enhance surgical precision, reduce fluoroscopy use and operative time, and improve implant positioning accuracy. Despite these advantages, wider clinical adoption remains limited by design time, production costs, and the need for regulatory standardization.

Extensive longitudinal researches are needed for understanding the life-span of these implants and their long-term function (Belvedere et al., 2019; Nagarajan et al., 2018). However, few studies exist to compare clinical results between 3D-printed and traditional implants because existing data is limited and inconsistent. The studies presented differences in fracture classification, implant composition, surgical practices, follow-up timeframes, and outcome assessment criteria. The potential benefits of PSI regarding aesthetic and technical results have been documented by some research studies; although other investigations reveal doubts about its additional worth next to standard methods due to financial and practical considerations (Jindal et al., 2021; Roelofs et al., 2024). Clinical practice and therapeutic guidelines require thorough evaluation of complication rates involving infection, nonunion, implant loosening, and articular step-off in both implant approaches (Alam, 2024).

This study systematically evaluates 3D-assisted fixation methods, with an emphasis on PSIs, while also considering related applications such as surgical guides and preoperative bone models, which represent complementary uses of the same 3D-printing platform. Upon reviewing the evidence this study aims to determine the optimal contexts for deploying 3D-printed implants. To the best of our knowledge and based on existing research in English publications no comprehensive review paper explored 3D printing applications in periarticular fractures. This study will promote evidence-based implementation of the technology by illuminating areas where additional research needs to occur like randomized trials and cost-effectiveness studies.

## 2 Methods

### 2.1 Literature search

A literature search was performed in PubMed, Scopus, and Web of Science from January 2015 to May 2025. The search strategy combined MeSH terms and free-text keywords including “3D printing,” “additive manufacturing,” “patient-specific implant,” “custom plate,” “surgical guide,” “fracture fixation,” “periarticular fracture,” “acetabular fracture,” “distal femur fracture,” “tibial plateau fracture,” and “distal radius fracture.” Boolean operators (AND, OR) were used to refine the query. The search strategy was developed based on the PICO framework, focusing on patients with complex periarticular fractures (P), comparing 3D-printed PSI or surgical guides (I) with conventional fixation methods (C), and assessing outcomes such as operative time, radiographic alignment, functional scores, and complications (O). The full PICO framework is shown in Table 1. Filters were applied to restrict results to English-language human clinical studies. We restricted our search to English language articles to minimize quality assessment bias; however, we are aware that studies, particularly from Asia where 3D printing for orthopedic trauma is emerging, that were not published in English may have been missed. Reference lists of included articles and related reviews were also screened to identify additional eligible studies.

**TABLE 1:** PICO framework for search strategy.

Component	Description
P (Population)	Patients with complex periarticular fractures, including tibial plateau fractures, distal femur fractures, acetabulum fractures, and distal radius fractures
I (Intervention)	Use of 3D-printed patient-specific implants, pre-contoured plates based on 3D models, or 3D-printed surgical guides/planning tools
C (Comparison)	Conventional fracture fixation techniques (standard precontoured plates, intraoperative plate bending, or conventional planning)
O (Outcomes)	Intraoperative outcomes (e.g., operative time, blood loss, fluoroscopy use), radiographic outcomes (e.g., articular step-off, alignment), fracture reduction, functional outcomes, complication rates, and time to union

The full electronic search strings used for all databases are provided below to ensure reproducibility:

PubMed: (“3D printing” OR “additive manufacturing” OR “patient-specific implant” OR “custom plate” OR “surgical guide”) AND (“fracture fixation” OR “periarticular fracture” OR “acetabular fracture” OR “distal femur fracture” OR “tibial plateau fracture” OR “distal radius fracture”).

Scopus: TITLE-ABS-KEY (“3D printing” OR “additive manufacturing” OR “patient-specific implant” OR “custom plate” OR “surgical guide”) AND TITLE-ABS-KEY (“fracture fixation” OR “periarticular fracture” OR “acetabular fracture” OR “distal femur fracture” OR “tibial plateau fracture” OR “distal radius fracture”).

Web of Science: (“3D printing” OR “additive manufacturing” OR “patient-specific implant” OR “custom plate” OR “surgical guide”) AND TS = (“fracture fixation” OR “periarticular fracture” OR “acetabular fracture” OR “distal femur fracture” OR “tibial plateau fracture” OR “distal radius fracture”).

### 2.2 Inclusion and exclusion criteria

Our selection process focused on human clinical studies such as randomized controlled trials (RCTs) and cohort studies which evaluated the effectiveness of 3D-printed PSIs compared to traditional methods for treating complex fractures around joints including distal femur and tibial plateau fractures. In this review, complex periarticular fractures refer to fractures occurring within roughly 5 cm of the joint surface, typically involving the metaphyseal or epimetaphyseal region, with comminution or articular incongruity requiring precise anatomical reduction (Natoli et al., 2019). Open fractures were excluded unless explicitly reported as Gustilo type I and managed with standard internal fixation protocols, as such cases may share similar surgical challenges to closed complex fractures. Studies had to report at least one of the following outcomes: intraoperative metrics (such as operative time and estimated blood loss), functional recovery, radiographic alignment, and/or complications. We only considered articles published in English from 2015 to 2025 to consider new evidence on 3D printing technologies. We excluded case reports, reviews, conference abstracts, editorials, animal, cadaveric or simulation-based studies, and studies with no comparator group using traditional fixation methods.

It should be noted that this review included studies evaluating various 3D-assisted techniques, including preoperative models, surgical guide templates, and PSIs. Because these represent different applications of the same underlying technology, the results were not quantitatively pooled but analyzed descriptively. Each category was discussed separately in the discussion section to minimize methodological bias arising from heterogeneity of interventions.

### 2.3 Study selection

All identified records were imported into EndNote (version X9) and duplicates were deleted. Two reviewers independently examined both titles and abstracts. Full-text articles underwent assessment for eligibility by applying inclusion and exclusion criteria. Conflicting decisions among reviewers were settled using consensus or with the assistance of an additional reviewer.

### 2.4 Data extraction

A standardized data extraction form was developed and piloted. Two reviewers conducted independent data extraction from included studies and any disagreements were resolved through discussion or consultation with a third reviewer. Extracted information was organized into two main tables:

- Study Characteristics Table: including authors, year, country, period, study design, sample size, fracture location, implant type, type of 3D intervention, surgical planning method, exclusion criteria, and follow-up duration.
- Outcome and Findings Table: including operative time, intraoperative blood loss, fluoroscopy time, time to union, complication rates, radiographic results, fracture reduction, cost evaluation, and main conclusion.

### 2.5 Risk of bias assessment

The methodological quality and risk of bias (ROB) were assessed using the ROBINS-I tool (Thomson et al., 2018). Five domains were rated: bias from confounding, selection of participants, missing data, deviations from intended interventions, and measurement of outcome. The potential ROB in each domain was judged as low, moderate, serious, or critical. The overall ROB for each study was based on the most serious ROB for each domain. The assessment was independently performed by two reviewers, with disagreements resolved through consensus.

## 3 Results

### 3.1 Study selection

**FIGURE 1:**
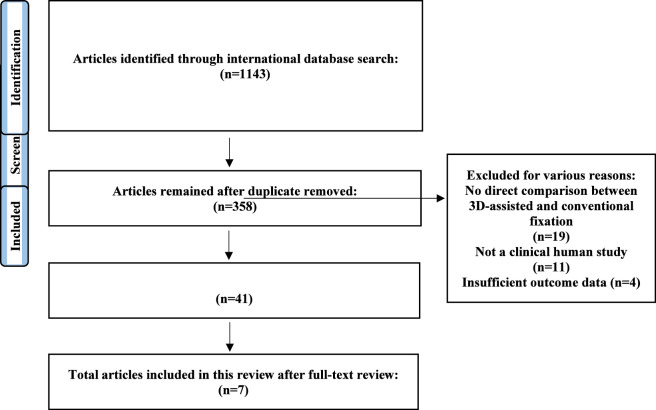
Flowchart of eligible studies included in this systematic review.

After conducting a comprehensive literature search across selected international databases, a total of 1143 studies were initially identified. Among these, we removed 785 duplicate records. From the remaining 358 studies, we screened the title and abstract and excluded 317 studies against the exclusion criteria. The full texts of 68 possibly relevant articles were evaluated for eligibility. Finally, 34 articles did not meet the inclusion criteria, and 7 studies were included in this review. The process of study selection is presented in the Preferred Reporting Items for Systematic Reviews (PRISMA) flow diagram in Figure 1.

### 3.2 Study characteristics

Our systematic review included seven articles from 2018 to 2024 based on established inclusion criteria. The analysis covered five RCTs alongside one prospective cohort study and one retrospective cohort study. The seven studies were from China, Spain, Korea, the Netherlands, and Bulgaria. The fracture locations analyzed were the acetabulum as well as the distal femur, tibial plateau, and distal radius. Sample sizes ranged from 20 to 70 patients, with comparative groups involving both 3D-printed PSIs or guides and conventional fixation methods. Five out of seven studies used patient specific implants. One study used a surgical guide/template and one study used a preoperative model for plate contouring. Most studies used CT or radiography-based planning techniques, with 3D printing applied either for plate pre-contouring, the creation of patient-specific implants (PSI), or patient-specific instrumentation such as drilling guides. Follow-up durations varied from 2 weeks to 18 months, with functional, radiographic, and intraoperative outcomes reported. A summary of study characteristics is presented in Table 2.

**TABLE 2:** Summary of study characteristics.

Author, ref	Year	Country	Period	Study design	Sample size		Fracture Site(s)	Type of 3D intervention	3D-printed implant type	Planning method	Exclusion criteria	Follow-up duration
					PSI	Conv						
Zhang (2018)	2018	China	Jan 2015 to August 2015	RCT	31	31	Femoral intercondylar	Patient-specific implant	LISS locking plate system	CT, Radiography	Age <18 years, open fractures, knee joint degeneration and injury previously, factures above a total knee arthroplasty, pathologic fracture, combined with systemic autoimmune disease	12 months
Wang et al. (2020)	2020	China	Jan 2016 to June 2017	RCT	15	35	Acetabulum	Patient-specific implant	Custom-shaped plate	CT	Age <18 or >65 years, open fractures, fractures involving the posterior wall	12 months
Ivanov et al. (2022)	2022	Bulgaria	Sep 2018 to December 2021	RCT	10	12	Acetabulum	Preoperative 3D model + implant planning	Custom-shaped plate	CT	NA	18 months
Giraldo (2023)	2023	Spain	May 2018 to November 2021	RCT	15	15	Distal radius and wrist	Patient-specific implant	Volar locking plate	CT, Radiography	Age <18 or >80 years, bilateral fractures, previous wrist fractures, non-displaced fractures, open fractures, inflammatory diseases affecting the wrists, fractures of more than 2 weeks’ duration, and associated fractures	15 months
Sakong et al. (2023)	2024	Korea	Jan 2019 to June 2020	RCT	9	11	Acetabulum	Preoperative 3D model for plate contouring	Locking plate	CT	Age <18 years, open fracture, peri implant fracture, old and pathologic fracture, and contralateral side fractures and dislocation	9 months
Assink et al. (2024)	2024	Netherlands	Jan 2021 to April 2023	Prospective cohort	15	15	Tibia	Surgical guide/template	3D-printed drills	CT	Age <18 years, open fractures, pathological fractures, or were treated nonoperatively	2 weeks
Duan et al. (2024)	2024	China	Sep2020 to January 2023	Retrospective cohort	22	22	Tibia and knee	Patient-specific implant (customized plate)	Customized steel plate	CT, Radiography	Age <25 or >60 years, Open fractures, severe concomitant injuries on the same limb, and patients with severe hepatic or renal dysfunction, cardiovascular or cerebrovascular diseases	12 months

### 3.3 Key data and outcome measures of included studies

Results from the studies show a wide variety of outcome measures that encompass intraoperative time duration, radiographic measurements, fracture reduction quality, functional recovery and complication monitoring. All the studies had comparative frameworks yet showed inconsistencies between fracture sites, implant designs, 3D printing applications, and follow-up periods leading to non-uniform outcome data across them.

Six studies provided data on operative time, five of which favored the 3D-assisted groups, indicating shorter duration. Zhang (2018) reported a mean duration of 61.2 min in the 3D group *versus* 79.1 min in the conventional group, while Duan et al. (2024) reported a decrease from 102 to 75 min. Similarly, intraoperative blood loss also appears to be decreased in the 3D groups in most of the included studies. Wang et al. (2020) reported an average blood loss of 880 mL (3D) *versus* 1177 mL (conventional). Intraoperative fluoroscopy frequency, another surrogate for surgical precision and confidence, was also lower in the 3D-assisted groups except one, which did not find any significant difference (Giraldo, 2023).

Radiographic evaluation of reduction quality, particularly articular step-off and residual gap, was reported in four studies. Ivanov *et al.* reported “anatomic” reduction in 80% of patients treated with 3D-guided planning *versus* only 50% in the control group. These findings suggest that PSIs or patient-specific instrumentation (guides and planning tools) contribute to more accurate anatomical reduction, which may influence long-term joint function. Radiographic follow-up parameters such as alignment, articular congruity, and time to union were variably reported. In general, time to union was comparable between groups, ranging from 13 to 18 weeks, with no significant delays in the 3D-assisted groups. However, overall radiographic congruency was slightly better in the 3D groups in studies that reported measurable displacement or angular correction.

Functional outcome was reported using HSS (Hospital for Special Surgery Knee Score), SFMA (Short Musculoskeletal Function Assessment), MSS (Matta Scoring System), PRWE (Patient-Rated Wrist Evaluation), and Merle d'Aubigné score. Zhang *et al.* found similar knee function scores between groups at 6 months and 12 months (Zhang, 2018). Ivanov *et al.* and Duan *et al.* reported moderately improved functional scores with 3D-assisted groups (Ivanov et al., 2022; Duan et al., 2024). Moreover, reported complication rates were also lower for 3D-assisted surgery. Most studies reported reduced complication rates in the 3D groups. Wang et al. (2020) observed only one case of screw loosening in the 3D group compared to multiple complications including infection, DVT, and nerve injury in the conventional group. Assink *et al.* reported a lower rate of screw misplacements with the application of patient-specific drills (Assink et al., 2024). Infections, malreduction, hardware failure, and neurovascular injury were the most commonly reported complications in the most studies. The incidence of these complications was either lower or comparable between the 3D-assisted groups. The reduced operative time, shorter fluoroscopy duration, and fewer complications reported in most 3D groups may imply potential long-term cost benefits, which warrant further investigation. Although not the primary focus, two studies briefly mentioned costs associated with 3D printing. Additional expenses ranged between $50 and $200 per case, covering printing and modeling. However, no study conducted a full cost-effectiveness analysis. The absence of detailed cost analyses across studies limits our understanding of the true economic value of 3D-assisted surgery. Although, reduced operative time and complication rates may imply downstream savings, this assumption cannot be validated without prospective, standardized cost-effectiveness evaluations that incorporate equipment, design, printing, and operating room costs.

In summary, 3D printing technology—whether applied through PSIs, surgical guides, or preoperative models—has shown to improve intraoperative efficiency and reduction accuracy. Improvements in functional outcomes, although limited, did show a small but consistent trend in favor of 3D-assisted techniques, with significant improvements seen in complex and joint-spanning fractures. Future well-designed randomized trials with larger sample sizes, as well as economic analysis are required to further assess these findings. A summary of key data and outcome measures is presented in Table 3.

**TABLE 3:** Summary of key data and outcome measures.

Operative time (min)		Blood loss (mL)		Fluoroscopy time (min)		Time to union (weeks)		Functional outcome score		Complication rate (%)		Radiographic implications	Fracture reduction quality		Cost evaluation for 3D-implant	Summary of main results	Ref
PSI	Conv	PSI	Conv	PSI	Conv	PSI	Conv	PSI	Conv	PSI	Conv		PSI	Conv			
61.2 ± 14.28	79.1 ± 18.4	81.7 ± 23.5	106.80 ± 22.7	2.10 ± 0.50	4.10 ± 0.70	NA		HSS, SFMA: 87.1%	80.6%	3.2%	9.7%	Fracture were in well reduction and fixation in PSI groups	NA		NA	3D-printed implants achieved less trauma, more accurately fixation and satisfactory recovery	Zhang (2018)
141.7 ± 52.9	170.7 ± 40.6	880.0 ± 673.4	1177.1 ± 691.6	NA		18 weeks for PSI		MSS: 66.7%	51.4%	6.6%	14.2%	PSI group had no reduction quality	No difference (*P* > 0.05)		NA	3D-printed implants were safe and effective for acetabular fracture more suitable for treatment of acetabular fractures	Wang et al. (2020)
193.5 ± 26.0	252.5 ± 30.2	665.0 ± 52.7	837.5 ± 68.0	NA		NA		Merle d’aubigne postel: 14.6	13.1	10%	25%	Accurate anatomical fit in PSI group	80% good reduction	50% good reduction	NA	3D-printed approach was associated with decreased operative time, less blood loss, and lower x-ray radiation	Ivanov et al. (2022)
No difference (*P* > 0.05)		NA		No difference (*P* > 0.05)		NA		PRWE: no difference (*P* > 0.05)		NA		No differences in radiological values, except the articular step (p = 0.028)	NA		NA	3D printing has not improved the parameters studied in relation to routinely operated patients	Giraldo (2023)
294.3	332.5	866.6	1040	NA		Gradual improvement after 3 days for PSI		NA		9%	None	Accurate anatomical fit in PSI group	NA		Additional expenses 50-100$ USD	3D-assisted precontoured plates allowed the surgeon to pre-contour the plate to the exact desired contour	Sakong et al. (2023)
NA		NA		NA		NA		NA		NA		Accurate anatomical fit in PSI group	3.1 mm	4.7 mm	Additional expenses 50-200$ USD	Use of 3D surgical planning including drilling guides was feasible, and facilitated accurate screw directions, screw lengths, and plate positioning	Assink et al. (2024)
75.2 ± 15.9	102.8 ± 21.1	129.3 ± 25.3	158.1 ± 33.6	The number of fluoroscopy sessions 6.5 ± 2	11.32 ± 3.5	13.4 ± 1.4	13.8 ± 1.6	HSS 90.5 ± 2.2	87.8 ± 2.8	9.1%	19%	Follow-up X-rays showed satisfactory fracture reduction and fixation	Satisfactory fracture reduction and fixation in PSI		NA	3D-printed implants can reduce surgical time, improve the accuracy of implant placement and matching, and facilitate postoperative joint function recovery	Duan et al. (2024)

### 3.4 Risk of bias assessment

**FIGURE 2:**
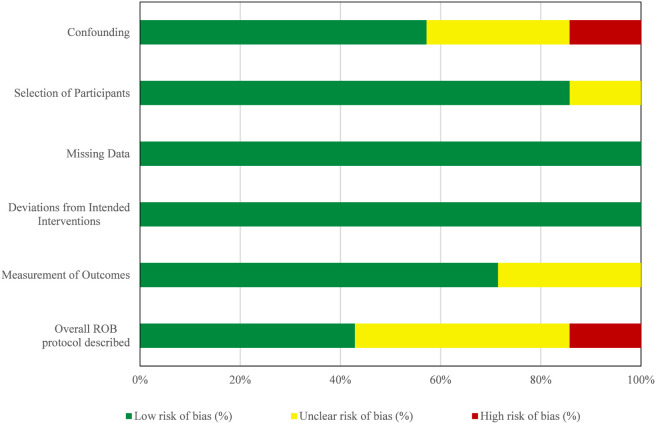
Distribution of risk of bias domains across all included studies.

ROB was assessed for all seven included studies using the ROBINS-I tool (Table 4; Figure 2). Of the seven studies included, five were RCTs and two were observational cohort studies (one prospective and one retrospective). As anticipated, the RCTs generally had lower ROB in most domains. In the domain of confounding, four studies had low risk ratings, while three studies had serious or moderate risk ratings based on small sample sizes, lack of adjustment for fracture severity, or use of non-concurrent control groups. Selection of participants was at low risk in all but one study (moderate in Assink *et al.*) where the control group was enrolled non-concurrently. Missing data was low across all studies indicating reporting of follow-up and completeness of outcome data was adequate. Overall deviations from intended interventions were low in all studies as there were no reports of any variation from study protocols as described in the methods sections of the reports. In terms of measurement of outcomes, the risk was considered low in most RCTs and moderate in one cohort study (Assink *et al.*) due to the inclusion of subjective or reported outcomes with limited blinding. The overall ROB was low in three studies (Zhang *et al.*, Sakong *et al.*, Giraldo *et al.*), moderate in three (Wang *et al.*, Ivanov *et al.*, Duan *et al.*) and serious in one (Assink *et al.*). The disparities in bias risk reveal study heterogeneity and the necessity for more robust well-powered comparative trials. Figure 2 graphically presents the distribution of ROB domains across all included studies.

**TABLE 4:** Risk of bias assessment.

Study	Confounding	Selection of participants	Missing data	Deviations from intended interventions	Measurement of outcomes	Overall ROB
Zhang (2018)	Low: no major baseline imbalance	Low: patients recruited pre-intervention	Low	Low: protocol followed	Low: objective measures (time, blood loss, ROM) used	Low
Wang et al. (2020)	Moderate, limited baseline adjustment info	Low	Low	Low: protocol followed	Low	Moderate
Ivanov et al. (2022)	Moderate, small sample, no adjustment for fracture severity	Low	Low	Low: protocol followed	Low	Moderate
Sakong et al. (2023)	Low: no major baseline imbalance	Low	Low	Low: protocol followed	Low	Low
Assink et al. (2024)	Serious, baseline imbalance	Moderate, groups not enrolled concurrently	Low, short-term data but seemingly complete	Low: protocol followed	Moderate, mixed objective/subjective outcomes	Serious
Giraldo (2023)	Low: no major baseline imbalance	Low, clear, prospective randomization	Low	Low: protocol followed	Low	Low
Duan et al. (2024)	Low: no major baseline imbalance	Low: clear grouping (3D vs. control) based on treatment	Low	Low: protocol followed	Moderate: unclear blinding	Moderate

## 4 Discussion

Surgeons regularly employ implants for periarticular fracture fixation but standard implants usually cause poor anatomical matching and longer surgical durations while causing difficult fracture reductions in complex or shattered injuries (Stavropoulos et al., 2019). Patient-specific surgical planning and implant design now have new possibilities because of rapid development in 3D printing technology (Attarilar et al., 2020; Popov et al., 2018). Through a systematic review of existing comparative research the authors evaluated the clinical effects of new surgical technologies. This systematic review aimed to compare the clinical, radiographic, and intraoperative outcomes of 3D-printed PSI and surgical tools with conventional fixation techniques in complex periarticular fractures. Across seven included studies involving distal femur, tibial plateau, acetabulum, and distal radius fractures, the findings suggest that 3D-assisted surgical approaches provide measurable advantages in operative efficiency and fracture reduction accuracy, while functional outcomes and complication rates remain comparable or improved.

### 4.1 Interpretation of main findings

The most frequently reported benefit across included studies was the reduction of operative time and intraoperative blood loss for the 3D-assisted groups. These improvements were likely due to improvements in preoperative planning, implant fit, and less intraoperative time for modifications. Both Duan et al. (2024) and Zhang (2018) found a significant reduction in operative time from 15 to 27 min less and Zhang (2018) found 3D-guided surgeries had up to 25 mL less intraoperative blood loss.

In this review, it is important to distinguish the benefits of different 3D printing applications, as they influence surgical outcomes in distinct ways. 3D-printed bone models mainly assist in preoperative planning, helping surgeons visualize fracture morphology and simulate reduction, which can shorten surgical time and improve accuracy of fixation. 3D-printed surgical guides or patient-specific instruments provide intraoperative precision, ensuring accurate drilling, screw placement, or osteotomy cuts, thereby minimizing fluoroscopy use and soft tissue disruption. In contrast, 3D-printed patient-specific implants directly impact postoperative outcomes by improving implant fit, load distribution, and joint congruity, which may enhance stability and reduce complications. Recognizing these distinctions clarifies that each 3D-assisted approach contributes at a different stage of the surgical workflow—from planning to execution to long-term recovery.

In the following discussion, we examine three distinct 3D-assisted methods—preoperative planning models, surgical guide templates, and PSIs—while emphasizing their respective clinical applications and surgical implications. It is important to note that, although these techniques share a common technological platform, they differ in clinical indications and surgical outcomes. Therefore, this review does not aim to directly compare these modalities or their clinical results. Instead, each will be discussed individually to highlight its specific advantages and limitations in the management of periarticular fractures.

3D-assisted approaches may lead to improved results when performing fracture reductions. In both Assink et al. (2024) and Ivanov et al. (2022), there was significantly less articular step-off in the 3D group and a higher rate of anatomic reduction, which could imply better joint-preserving results and long-term functionality. These findings could be a testimony to the concept that patient-specific instruments allow for greater accuracy in the complex anatomy of acetabulum or tibial plateau region. Moreover, the scores of functional outcomes, such as HSS, PRWE, and Merle d'Aubigné scores, were generally better in the 3D groups. However, these differences were small and sometimes statistically insignificant (Giraldo, 2023). This may be due to the impact of patient-related factors (such as age and comorbidities) and rehabilitation differences which could obscure the effect of accuracy of surgery on functional outcomes. However, no studies reported worse functional outcome scores in the 3D group. Also, most studies reported that the rate of complication is lower or similar in the 3D-assisted groups. In theory, PSI could prevent hardware malposition, allow better accuracy of screw placement, and reduce the risk of revision. The results might be limited by the small sample sizes of included studies.

The three most reported outcomes, where clear superiority of 3D-assisted fixation was identified, were the following: the reduction of surgical time was found in majority of the studies included in this review, and this result can be explained by the preoperative time-saved during surgery planning and the intraoperative time-saved due to less adjustment of the implant to the bone. The 3D printing technology allowed complete elimination or significant reduction of step-offs of joint surfaces after reduction of fracture, as well as, in several studies, significantly decreased postoperative complications (infection, misplaced hardware, malreduction) due to the improved accuracy and reduced soft-tissue dissection during the procedure. In summary, it can be concluded that the main benefit of 3D printing is in reducing intraoperative morbidity and improving the accuracy of the procedure.

It is worth noting that one of the included studies, Assink et al. (2024), found no difference in clinical or radiographic outcomes between 3D-assisted and conventional treatment groups. This discrepancy may be due to a number of reasons. Methodologically, it should first be noted that their use of a historical control group would have raised the risks of selection bias and temporal confounding. The study sample was also small (15 patients), which could have resulted in inadequate power. Also, the outcome measures were limited to the early postoperative period; if the study had an adequate length of follow-up, it is possible that 3D-assisted and non-3D-assisted treatment would not differ with respect to functional outcomes in the short term. Furthermore, no intraoperative metrics (surgical time, blood loss, etc.) were measured, which is unfortunate, since these represent some of the domains where other studies have found the clearest benefit of 3D-guided intervention.

When examined by application type, certain trends were observed across studies. 3D-printed preoperative models were particularly valuable in fractures with complex morphology (e.g., acetabular and distal femur), where visualizing fracture geometry improved surgical planning and plate contouring. Surgical guide templates showed the greatest intraoperative benefit, notably in reducing operative time and fluoroscopy use in tibial plateau fractures. Meanwhile, PSIs offered superior anatomical fit and were associated with more accurate reduction in articular fractures, especially of the acetabulum and distal radius. Although these findings are qualitative, they suggest that 3D models enhance planning, templates improve precision during surgery, and implants provide postoperative structural advantages.

### 4.2 Preclinical and review evidence

Several recent experimental studies on non-human models support the theoretical advantages of 3D-printed custom implants, even though they were outside our inclusion criteria. For example, Baumgartner et al. (2023) reported that a generative-designed 3D-printed mandible plate in dogs could be perfectly aligned to the bone surface (“avoid critical structures, achieve perfect alignment to individual bone contours”) and demonstrated 2.8–3.6× higher ultimate strength than standard designs. In a related cadaveric study, patient-specific titanium mandibular cage implants showed no hardware failures under quasi-static loading and were deemed to have “satisfactory mechanical functioning under demanding chewing conditions” (van Kootwijk et al., 2023). Likewise, a finite-element analysis of a large distal femur defect found that a custom 3D-printed titanium implant provided a stable fixation with “excellent lightweight structure” and broad bone contact areas for ingrowth (Wong et al., 2020). Although these animal and bench models differ from clinical fractures, they reinforce that patient-specific designs can achieve secure bone-implant fit and adequate strength that align with the implant accuracy and stability we infer from human outcomes.

In contrast, recent literature reviews have generally assessed 3D-printed implants across a broad range of conditions, highlighting promising yet preliminary findings. For instance, Roy *et al.* found that 3D-printed custom implants afforded a highly personalized fit with reduced intraoperative time and complications (Roy et al., 2025). Ling *et al.* similarly observed that 3D-assisted orthopedic trauma care can shorten operations, decrease blood loss, and improve fracture reduction quality (Ling et al., 2025). McAnena *et al.* noted that patient-specific devices are promising for complex anatomy but stressed that “large controlled studies are necessary” to validate their safety and efficacy (McAnena et al., 2025). These reviews span many anatomical sites (spine, pelvis, maxillofacial, etc.) and often synthesize heterogeneous case reports or small series. Our systematic review contributes new evidence by focusing purely on periarticular fractures in actual patients. We directly compare several important parameters such as surgical time, radiographic alignment, union and complication rates, and functional scores, which were only indirectly related in previous reviews. In brief, while previous literature can generally support the potential of 3D-printed implants, the present study was the first to demonstrate the actual clinical impact of 3D-printed implants on surgical efficiency and patient outcomes in complex periarticular trauma, which was not covered by previous reviews.

### 4.3 Practical viability

It is important to note that the translation of 3D-assisted fixation techniques into the clinical setting not only requires excellent technical performance, but also a certain level of pragmatic feasibility in both routine and emergency practice. As it stands, with a general time for 3D-printed implant production of 24–72 h for image processing, design, and fabrication, the use of such technology is at present more suited to elective or semi-urgent surgical cases than primary acute trauma care fixation. Reported 3D printing costs in the included studies ranged from USD 50 to 200 per case, accounting primarily for design and printing material costs, which while relatively small in comparison to the total surgical cost, can still be prohibitive for use in low-resource settings without in-house 3D printing capabilities. In addition, 3D-assisted approaches can require close collaboration and communication between engineers, radiologists, and surgeons, which may not be easily or immediately replicated in an acute trauma setting. Therefore, the authors acknowledge that the practical viability is currently highest in planned or reconstructive fracture surgeries. Though it is likely to change in the near future with the advent of rapid prototyping technology and the growing accessibility of hospital-based 3D printing.

### 4.4 Limitations and recommendations for future research

Some limitations need to be considered. First, the small number of included studies (n = 7) and the limited sample size of these studies (usually 20–70 patients per study) do not provide sufficient statistical power or external validity to the analyses. Second, the significant heterogeneity of fracture sites (acetabulum, distal femur, tibial plateau, and distal radius), 3D interventions (patient-specific implants, guide templates, and preoperative models), and outcome measures precluded comparisons and generalizations across the included studies. Therefore, the pooled evidence presented in this study should be viewed with caution and until validated by larger, standardized, multicenter clinical trials. Third, while ROB was low for several RCTs, some cohort studies did not adjust for all domains, which may have resulted in moderate-to-serious ROB. Fourth, the follow-up periods between the involved studies were highly inconsistent, with a minimum of 2 weeks and a maximum of 18 months. In particular, Assink et al. (2024) only had a 2-week follow-up. This is far from enough time to assess bone union or the duration of the implant as well as the stability of functional improvement. Research protocols require follow-up evaluations to be established consistently between 6 and 12 months to enable proper assessment of healing progression. Finally, reporting of economic outcomes was poor and no cost-effectiveness analysis was available to balance the benefits of 3D technology against increased resource utilization. Two studies provided indirect data on the direct costs of 3D printing (USD 50–200 per case), while no study performed a formal cost–benefit or cost–utility analysis. Thus, the economic feasibility of 3D-assisted fixation remains to be established and should be prospectively evaluated in multicenter trials.

Research is needed to conduct RCTs that compare both 3D-printed and conventional fixation methods. These trials should be powered and multicentric with evaluations at different anatomical sites with standardized metrics and longer follow-up duration. There is a need to focus on the cost-effectiveness of the technology, the learning curve associated with the technology, and feasibility of 3D printing in acute trauma/emergency settings with time-sensitive decision-making.

## 5 Conclusion

This review demonstrates that patient-specific 3D-printed implants may improve operative efficiency and fracture reduction accuracy in complex periarticular fractures. Outcome measures such as operative time, radiographic criteria for fracture reduction quality, and complications in selected cases were more favorable for 3D-assisted fracture fixation and show potential advantages over standard of care fixation. However, due to limited and heterogeneous evidence, functional outcomes appear largely comparable between groups. PSIs may provide improved reduction and fixation in complex fractures, but the optimal role of 3D technology in routine orthopedic trauma surgery has yet to be determined and should be confirmed with additional large randomized trials and cost-effectiveness analyses.

## References

[B1] Alam M. I. (2024). 3D-Printed medical implants: recent trends and challenges, 1–21.

[B2] Ariz A. Tasneem I. Bharti D. Vaish A. Haleem A. Javaid M. (2021). Is additive manufacturing of patient-specific implant beneficial for orthopedics. Apollo Med. 18 (1), 33–40. 10.4103/am.am_20_20

[B3] Assink N. ten Duis K. de Vries J. P. P. M. Witjes M. J. H. Kraeima J. Doornberg J. N. (2024). 3D surgical planning including patient-specific drilling guides for tibial Plateau fractures: a prospective feasibility study. Bone Jt. Open 5 (1), 46–52. 10.1302/2633-1462.51.bjo-2023-0130.r1 38240277 PMC10797644

[B4] Attarilar S. Ebrahimi M. Djavanroodi F. Fu Y. Wang L. Yang J. (2020). 3D printing technologies in metallic implants: a thematic review on the techniques and procedures. Int. J. Bioprinting 7 (1), 306. 10.18063/ijb.v7i1.306 33585711 PMC7875061

[B5] Balamurugan P. Selvakumar N. (2021). Development of patient specific dental implant using 3D printing. J. Ambient Intell. Humaniz. Comput. 12 (3), 3549–3558. 10.1007/s12652-020-02758-6

[B6] Ballard D. H. Mills P. Duszak R. Jr. Weisman J. A. Rybicki F. J. Woodard P. K. (2020). Medical 3D printing cost-savings in orthopedic and maxillofacial surgery: cost analysis of operating room time saved with 3D printed anatomic models and surgical guides. Acad. Radiol. 27 (8), 1103–1113. 10.1016/j.acra.2019.08.011 31542197 PMC7078060

[B7] Baumgartner D. Schramel J. P. Kau S. Unger E. Oberoi G. Peham C. (2023). 3D printed plates based on generative design biomechanically outperform manual digital fitting and conventional systems printed in photopolymers in bridging mandibular bone defects of critical size in dogs. Front. Vet. Sci. 10, 1165689. 10.3389/fvets.2023.1165689 37065217 PMC10098091

[B8] Beheshtizadeh N. Azami M. Abbasi H. Farzin A. (2022). Applying extrusion-based 3D printing technique accelerates fabricating complex biphasic calcium phosphate-based scaffolds for bone tissue regeneration. J. Adv. Res. 40, 69–94. 10.1016/j.jare.2021.12.012 36100335 PMC9481949

[B9] Bell C. K. Edelhoff D. Prandtner O. Pour R. (2018). Accuracy of implants placed with surgical guides: thermoplastic *versus* 3D printed. Int. J. Periodontics Restor. Dent. 38 (1), 121–126. 10.11607/prd.2874 29240212

[B10] Belvedere C. Siegler S. Fortunato A. Caravaggi P. Liverani E. Durante S. (2019). New comprehensive procedure for custom‐made total ankle replacements: medical imaging, joint modeling, prosthesis design, and 3D printing. J. Orthop. Res. 37 (3), 760–768. 10.1002/jor.24198 30537247

[B11] Bertrand M. L. Andrés-Cano P. Pascual-López F. J. (2015). Periarticular fractures of the knee in polytrauma patients. Open Orthop. J. 9, 332–346. 10.2174/1874325001509010332 26312118 PMC4541416

[B12] Chai Y. Chen X. B. Estoque J. A. Birbilis N. Qin Q. Ward T. (2023). A novel approach of customized pelvic implant design based on symmetrical analysis and 3D printing. 3D Print. Addit. Manuf. 10 (5), 984–991. 10.1089/3dp.2021.0121 37886407 PMC10599429

[B13] Crowe C. S. Kakar S. J. (2023). Periarticular distal radius fractures and complex ligamentous injury: the role of arthroscopic evaluation. J. Orthop. Chestert. 42, 6–12. 10.1016/j.jor.2023.06.006 37389206 PMC10302116

[B14] Dei Giudici L. Faini A. Garro L. Tucciarone A. Gigante A. (2016). Arthroscopic management of articular and peri-articular fractures of the upper limb. EFORT Open Reviews 1 (9), 325–331. 10.1302/2058-5241.1.160016 28461964 PMC5367527

[B15] Duan S. Xu R. Liang H. Sun M. Liu H. Zhou X. (2024). Study on the efficacy of 3D printing technology combined with customized plates for the treatment of complex tibial Plateau fractures. J. Orthop. Surg. Res. 19 (1), 562. 10.1186/s13018-024-05051-w 39267139 PMC11391824

[B16] Einafshar M. M. Rajaeirad M. J. A. B. C. M. (2025). Design, manufacturing, patient-specific implant (PSI) by additive manufacturing. 22: p. 249.

[B17] Faghani-Eskandarkolaei P. Heli H. Akbari N. Koohi-Hosseinabadi O. Sari Aslani F. Sattarahmady N. (2024). Antibacterial and anti-biofilm activities of gold-curcumin nanohybrids and its polydopamine form upon photo-sonotherapy of *Staphylococcus aureus* infected implants: *in vitro* and animal model studies. Int. J. Biol. Macromol. 282, 137430. 10.1016/j.ijbiomac.2024.137430 39528199

[B18] Giraldo P. (2023). Randomized clinical trial on the usefulness of 3d printing in intra-articular fractures of the distal radius, S1888–S4415. 10.1016/j.recot.2024.12.002 39653141

[B19] Ivanov S. Valchanov P. Hristov S. Veselinov D. Gueorguiev B. (2022). Management of complex acetabular fractures by using 3D printed models. Medicina 58 (12), 1854. 10.3390/medicina58121854 36557056 PMC9785751

[B20] Jindal S. Manzoor F. Haslam N. Mancuso E. (2021). 3D printed composite materials for craniofacial implants: current concepts, challenges and future directions. Int. J. Adv. Manuf. Technol. 112 (3), 635–653. 10.1007/s00170-020-06397-1

[B21] Kim M. J. Lee H. B. Ha S. K. Lim D. J. Kim S. D. (2021). Predictive factors of surgical site infection following cranioplasty: a study including 3D printed implants. Front. Neurol. 12, 745575. 10.3389/fneur.2021.745575 34795630 PMC8592932

[B22] Ling K. Wang W. Liu J. (2025). Current developments in 3D printing technology for orthopedic trauma: a review. Med. Baltim. 104 (12), e41946. 10.1097/md.0000000000041946 40128051 PMC11936578

[B23] Luenam S. Kosiyatrakul A. Phakdeewisetkul K. Puncreobutr C. (2020). The patient-specific implant created with 3D printing technology in treatment of a severe open distal humerus fracture with complete loss of the lateral column. J. Orthop. Surg. Hong. Kong. 28 (3), 2309499020960251. 10.1177/2309499020960251 33021150

[B24] McAnena A. P. McClennen T. Zheng H. (2025). Patient-specific 3-Dimensional-Printed orthopedic implants and surgical devices are potential alternatives to conventional technology but require additional characterization. Clin. Orthop. Surg. 17 (1), 1–15. 10.4055/cios23294 39912074 PMC11791502

[B25] Moiduddin K. Mian S. H. Umer U. Alkhalefah H. Ahmed F. Hashmi F. H. (2023). Design, analysis, and 3D printing of a patient-specific polyetheretherketone implant for the reconstruction of zygomatic deformities. Polym. (Basel). 15 (4), 886. 10.3390/polym15040886 36850170 PMC9962529

[B26] Nagarajan N. Dupret-Bories A. Karabulut E. Zorlutuna P. Vrana N. E. (2018). Enabling personalized implant and controllable biosystem development through 3D printing. Biotechnol. Adv. 36 (2), 521–533. 10.1016/j.biotechadv.2018.02.004 29428560

[B27] Natoli R. M. Sardesai N. Richard R. Sorkin A. Gaski G. Virkus W. (2019). Intramedullary nailing of lower-extremity periarticular fractures. JBJS Essent. Surg. Tech. 9 (4), e35. 10.2106/jbjs.st.18.00112 32051781 PMC6974312

[B28] Onică N. Budală D. G. Baciu E. R. Onică C. A. Gelețu G. L. Murariu A. (2024). Long-term clinical outcomes of 3D-printed subperiosteal titanium implants: a 6-year follow-up. J. Pers. Med. 14 (5), 541. 10.3390/jpm14050541 38793123 PMC11122366

[B29] Patel R. McCarthy K. Christensen J. Jacobs B. Karsch J. Sephien A. (2024). Cost analysis and clinical outcomes of anatomic pre-contoured locking *versus* conventional plates for distal fibula ankle fractures. Eur. J. Orthop. Surg. Traumatol. 34 (2), 959–965. 10.1007/s00590-023-03728-2 37779131

[B30] Popov V. V. Muller-Kamskii G. Kovalevsky A. Dzhenzhera G. Strokin E. Kolomiets A. (2018). Design and 3D-printing of titanium bone implants: brief review of approach and clinical cases. Biomed. Engineering Letters 8, 337–344. 10.1007/s13534-018-0080-5 30603218 PMC6209081

[B31] Prządka M. Pająk W. Kleinrok J. Pec J. Michno K. Karpiński R. (2025). Advances in 3D printing applications for personalized orthopedic surgery: from anatomical modeling to patient-specific implants. J. Clin. Med. 14 (11), 3989. 10.3390/jcm14113989 40507750 PMC12156138

[B32] Roelofs L. J. Assink N. Kraeima J. ten Duis K. Doornberg J. N. de Vries J. P. P. M. (2024). Clinical application of 3D-Assisted surgery techniques in treatment of intra-articular distal radius fractures: a systematic review in 718 patients. J. Clin. Med. 13 (23), 7296. 10.3390/jcm13237296 39685754 PMC11642203

[B33] Rossi S. M. P. Andriollo L. Sangaletti R. Montagna A. Benazzo F. (2025). International, consensus-based, indications and treatment options for knee arthroplasty in acute fractures around the knee. Arch. Orthop. Trauma Surg. 145 (1), 154. 10.1007/s00402-025-05755-6 39891727

[B34] Roy M. Jeyaraman M. Jeyaraman N. Sahu A. Bharadwaj S. Jayan A. K. (2025). Evaluating effectiveness, safety, and patient outcomes of 3D printing in orthopedic implant design and customization: a PRISMA-complaint systematic review. J. Orthop. Case Rep. 15 (6), 213–222. 10.13107/jocr.2025.v15.i06.5720 40520756 PMC12159611

[B35] Sakong S.-y. Cho J. W. Kim B. S. Park S. J. Lim E. J. Oh J. K. (2023). The clinical efficacy of contouring periarticular plates on a 3D printed bone model. J. Pers. Med. 13 (7), 1145. 10.3390/jpm13071145 37511758 PMC10381594

[B36] Sattarahmady N. Kayani Z. Heli H. Faghani-Eskandarkolaei P. Haghighi H. (2024). Photosensitizing activity of nanoparticles of poly (2-amino Phenol)/gold for intensified doxorubicin therapeutic effect on melanoma cancer cells under synergism effect of 808-nm light. J. Biomed. Phys. Eng. 14 (6), 547–560. 10.31661/jbpe.v0i0.2312-1693 39726887 PMC11668927

[B37] Stavropoulos A. Bertl K. Eren S. Gotfredsen K. (2019). Mechanical and biological complications after implantoplasty—A systematic review. Clin. Oral Implants Res. 30 (9), 833–848. 10.1111/clr.416_13509 31254417

[B38] Thomson H. Craig P. Hilton-Boon M. Campbell M. Katikireddi S. V. (2018). Applying the ROBINS-I tool to natural experiments: an example from public health. Syst. Rev. 7, 15. 10.1186/s13643-017-0659-4 29368630 PMC5784724

[B39] Tomaževič M. Kristan A. Kamath A. F. Cimerman M. (2021). 3D printing of implants for patient-specific acetabular fracture fixation: an experimental study. Eur. J. Trauma Emerg. Surg. 47, 1297–1305. 10.1007/s00068-019-01241-y 31641786

[B40] van Kootwijk A. Jonker B. Wolvius E. Saldivar M. C. Leeflang M. Zhou J. (2023). Biomechanical evaluation of additively manufactured patient-specific mandibular cage implants designed with a semi-automated workflow: a cadaveric and retrospective case study. J. Mech. Behav. Biomed. Mater. 146, 106097. 10.1016/j.jmbbm.2023.106097 37678107

[B41] Wang C. Chen Y. Wang L. Wang D. Gu C. Lin X. (2020). Three-dimensional printing of patient-specific plates for the treatment of acetabular fractures involving quadrilateral plate disruption. BMC Musculoskelet. Disord. 21, 451–459. 10.1186/s12891-020-03370-7 32650750 PMC7350601

[B42] Willemsen K. Nizak R. Noordmans H. J. Castelein R. M. Weinans H. Kruyt M. C. (2019). Challenges in the design and regulatory approval of 3D-printed surgical implants: a two-case series. Lancet Digit. Health 1 (4), e163–e171. 10.1016/s2589-7500(19)30067-6 33323186

[B43] Wong K. W. Wu C. D. Chien C. S. Lee C. W. Yang T. H. Lin C. L. (2020). Patient-specific 3-Dimensional printing titanium implant biomechanical evaluation for complex distal femoral open fracture reconstruction with segmental large bone defect: a nonlinear finite element analysis. Appl. Sci. 10 (12), 4098. 10.3390/app10124098

[B44] Zhang R. (2018). Clinical effect of three-dimensional printing assisted less invasive stabilization system (LISS) to treat femoral intercondylar fracture. Int. J. Clin. Exp. Med. 11 (10), 10809–10814.

